# Evaluation of forces produced by therapeutic elastic resistance bands of various lengths and stiffnesses: a biomechanical study for clinical application

**DOI:** 10.3389/fvets.2026.1799600

**Published:** 2026-04-10

**Authors:** Phillip Saint-Martin, Daniel McCarthy, Pierre-Yves Mulon, Darryl Millis

**Affiliations:** 1 SACS Department, The University of Tennessee College of Veterinary Medicine, Knoxville, TN, United States; 2 CARES Center, The University of Tennessee College of Veterinary Medicine, Knoxville, TN, United States; 3 LACS Department, The University of Tennessee College of Veterinary Medicine, Knoxville, TN, United States

**Keywords:** biomechanics, elastic resistance band, rehabilitation, therapeutic band, veterinary physical rehabilitation, surgery

## Abstract

**Introduction:**

Elastic resistance bands (ERBs) are commonly incorporated into canine and human strengthening exercises to restore muscle strength during rehabilitation; however, there is limited published quantitative data regarding force production under conditions used in veterinary medicine. The objective of this study was to quantify the tensile forces produced by ERBs of different stiffnesses and lengths at various levels of elongation to generate specific guidelines for rehabilitation prescription of canine patients. We hypothesized that greater tensile forces would be generated by using stiffer ERBs, shorter resting lengths and greater elongation lengths.

**Methods:**

Six color-coded ERBs (THERABAND^®^) were evaluated at 10 cm and 40 cm resting lengths, with the aid of an Instron mechanical tester. Each band was stretched to 1.25x, 1.5x, and 1.75x their original resting lengths. 10 cm ERBs were stretched to 12.5 cm, 15 cm, and 17.5 cm respectively. 40 cm ERBs were stretched to 50 cm, 60 cm, and 70 cm, respectively. Mean peak forces were compared by one-way ANOVA with Tukey post hoc testing.

**Results and discussion:**

Forces increased linearly with elongation and ERB stiffness 26 (indicated by color of the band), with *R*^2^ values > 0.95 for each band color. 10 cm bands produced significantly higher mean forces than bands of 40 cm in length (*p* < 0.0001). Black bands generated the highest resistance across all elongation levels. Band stiffness, elongation, and starting length are key determinants of force generation. The quantification of these parameters enables the prescription of ERB exercises as part of a specific and repeatable rehabilitation dosing intervention in veterinary rehabilitation.

## 1 Introduction

Elastic resistance bands (ERBs) are widely used in both human and veterinary rehabilitation to enhance muscle strength, improve mobility, and aid recovery following injury or surgery (1–3). ERBs are widely used in human rehabilitation programs to improve strength and functional recovery following musculoskeletal injury and surgery (3–9). Similar resistance-based exercises are increasingly incorporated into veterinary rehabilitation programs for dogs recovering from orthopedic or neurological disease (3).

**Figure 1:**
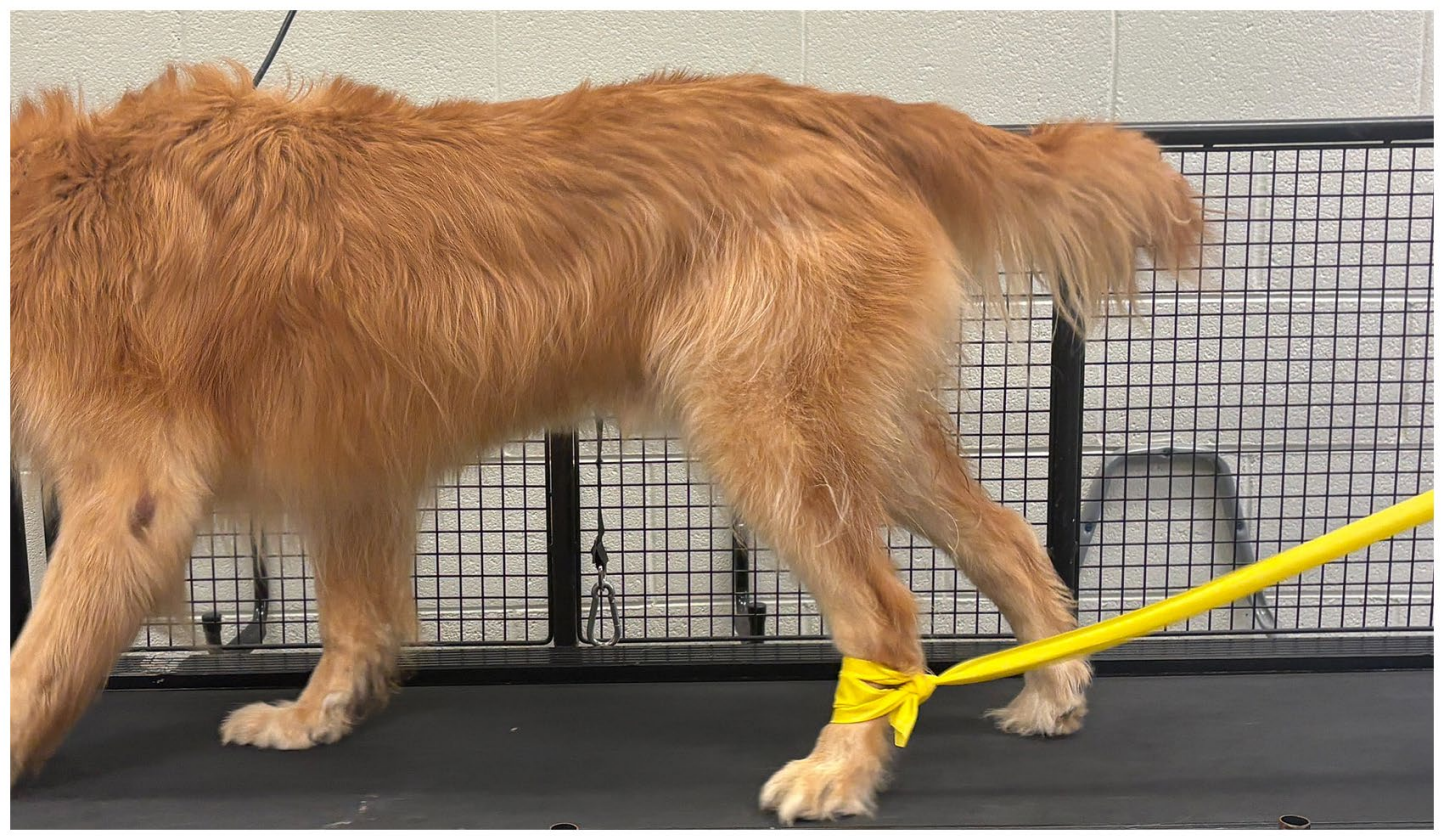
Example of THERABAND^®^ exercise in canine patient.

In veterinary rehabilitation, resistance exercises using elastic bands are commonly prescribed to improve limb strength, neuromuscular control, and functional recovery following orthopedic surgery or neurologic injury (Figure 1) (1, 9). Despite their frequent clinical use, the mechanical resistance produced by these bands under conditions commonly applied to canine patients remains poorly quantified (9). As a result, clinicians often prescribe resistance empirically without objective information regarding the forces applied to the limb (1). It is therefore imperative to have quantitative data establishing a link between band elongation and band color (as a proxy for stiffness) with the amount of force generated so that rehabilitation dosing can be standardized, consistent, and repeatable.

Most current studies evaluating ERB mechanical properties have been conducted in human rehabilitation, where exercise parameters and biomechanical demand differ from those in canine patients. Previous human studies have characterized ERB tension to standardized elongation levels (such as 100 and 200% increases in length); however, biomechanical differences between humans and dogs may significantly influence how elastic resistance is applied during rehabilitation exercises (10). Dogs are quadrupeds with different loading patterns, stride lengths, and joint excursion compared to humans (11). During locomotion, canine limb joints undergo different ranges of motion and operate under distinct ground reaction force distributions relative to bipedal human gait (12, 13). These differences may alter how resistance applied by elastic bands translates into joint moments and muscular loading during therapeutic exercises. Consequently, resistance values derived from human rehabilitation studies may not accurately reflect the mechanical loading experienced by canine patients, emphasizing the need for species-specific characterization of ERB force generation.

The objectives of the present study were therefore (i) to quantify the tensile forces produced by ERBs of varying stiffness (color-coded) at elongation levels of 1.25×, 1.5×, and 1.75× their resting lengths, and (ii) to compare the effects of two commonly used resting band lengths (10 cm and 40 cm) on forces generated during loading. We hypothesized that the ERBs would exhibit a linear force–elongation relationship, and that shorter, stiffer bands and greater elongation would produce proportionally higher tensile forces. Based on the data collected, developing guidelines for specific forces generated with different ERBs, starting band lengths and elongation lengths was an additional goal of this study. This manuscript is an expansion from preliminary data reported in 2019 (2).

## 2 Materials and methods

**Figure 2:**
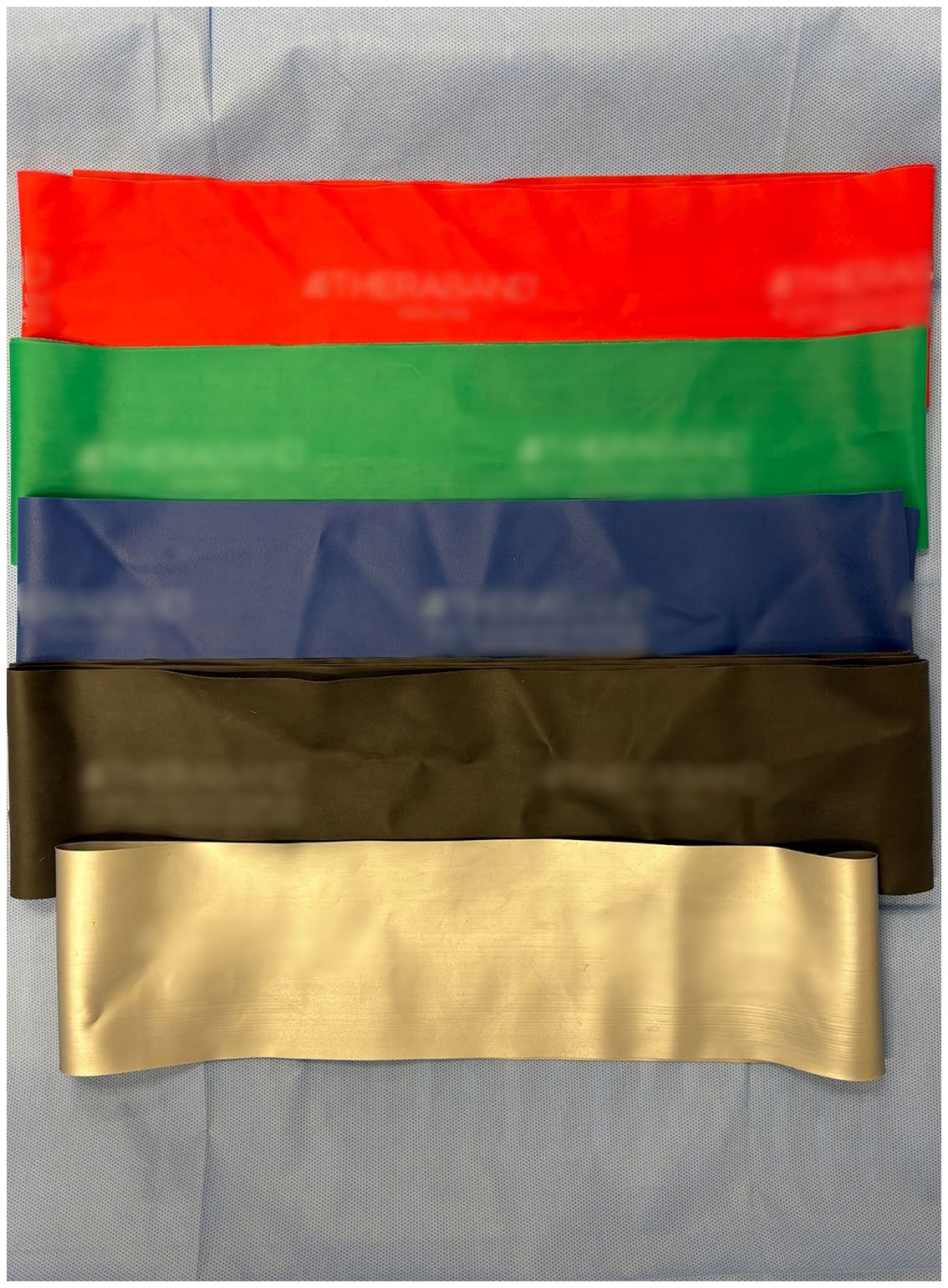
THERABAND^®^ example colors.

**Figure 3:**
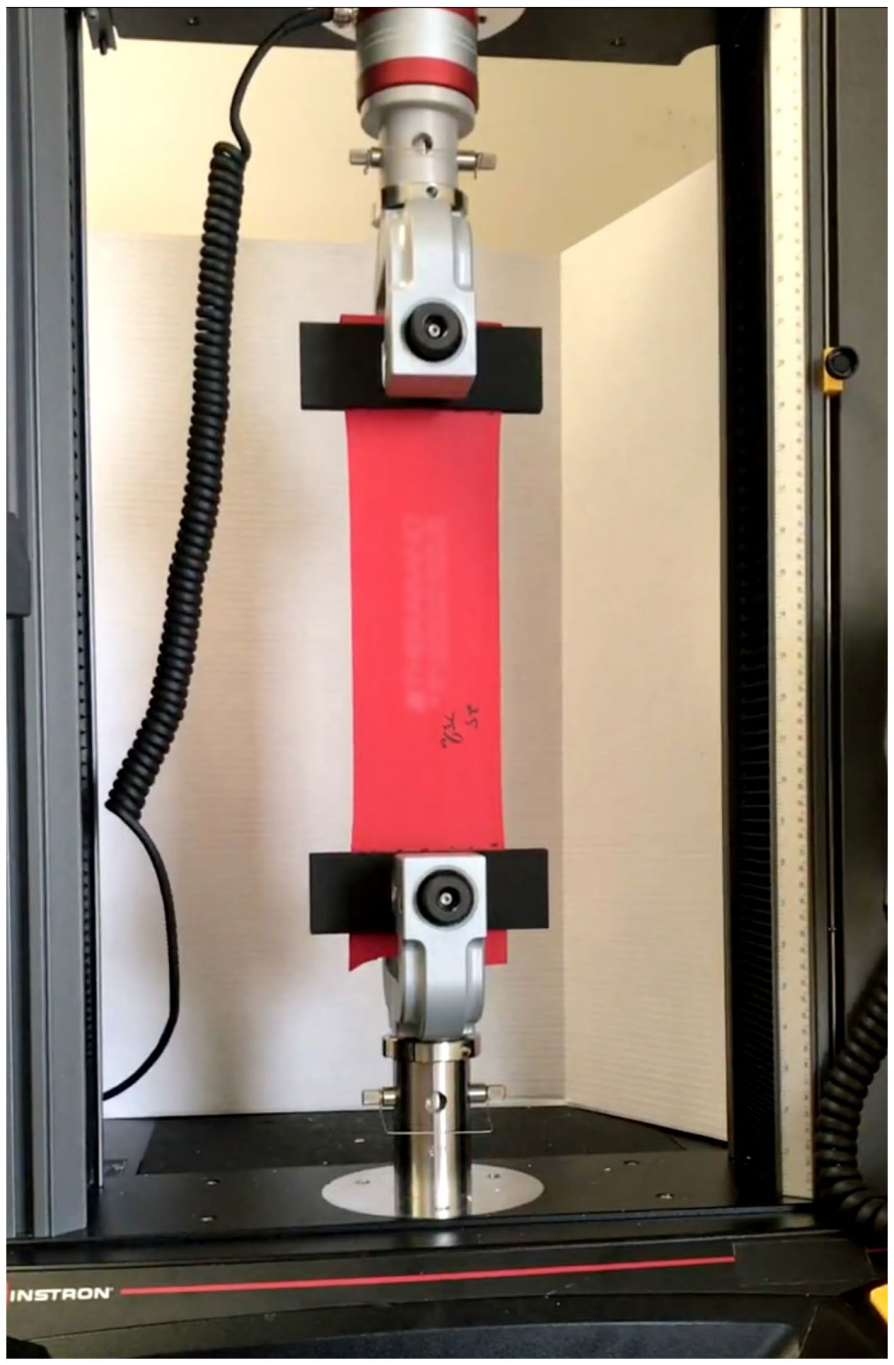
THERABAND^®^ loaded onto Instron biomechanical testing device.

Six color-coded ERBs (tan, yellow, red, green, blue, and black; see Figure 2) were evaluated, with colors organized according to ascending stiffness (THERABAND^®^, Akron, OH, USA) (10). Five separate bands of each color were prepared at two resting lengths (10 cm and 40 cm) for each elongation length and mounted on an Instron Biomechanical Testing Device (Model 5969, Instron, Norwood, MA, USA). The chosen lengths were limited by the biomechanical testing device, especially considering the elongation lengths tested. The Instron system was calibrated using a 50 N load cell, and ambient temperature was maintained at 22 ± 1 °C throughout testing. Bands were secured with non-slip pneumatic grips with a 2 cm overlap to prevent slippage as seen in Figure 3. Prior to testing, each band was preconditioned with 10 loading cycles to minimize viscoelastic hysteresis (14).

**Figure 4:**
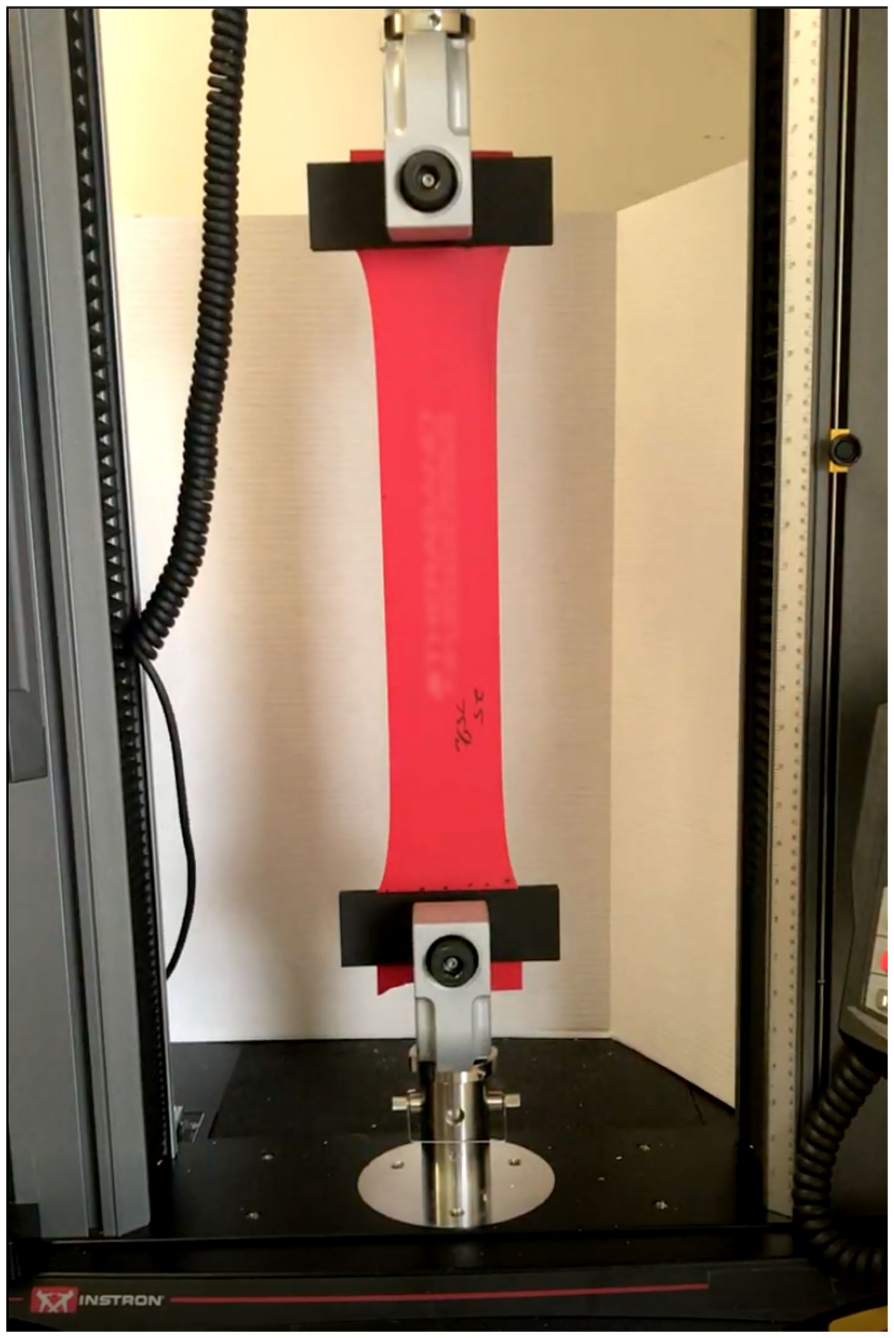
Theraband stretched using the Instron biomechanical testing device.

For each test condition (resting length and elongation length), the maximal force generated during each elongation cycle was recorded. Each band was elongated at a constant rate of 3 m/min for 30 consecutive cycles to three elongation levels (1.25×, 1.5×, and 1.75 × of the original resting length) using newly preconditioned bands for each length (Figure 4). The elongation rate was selected to provide consistent loading conditions while minimizing viscoelastic artifacts associated with variable stretching speeds (14).

Five replicates were tested for each combination of band color, resting length, and elongation level. The mean peak force across the 30 loading cycles was calculated for each band replicate. The resulting mean peak force values were then averaged across the five bands to generate the reported mean ± standard deviation values for each condition. Force was measured and analyzed in Newtons (N), the SI unit of force. For clinical interpretation, equivalent values expressed in kilograms of force (kg), were also reported in selected figures and tables. Force–elongation data were collected using Bluehill software (Instron, Norwood, MA, USA) and exported for statistical analysis.

One-way analysis of variance (ANOVA) was used to compare mean peak forces among ERB colors at each elongation level separately for the two resting band lengths (10 cm and 40 cm). When a significant effect was detected, Tukey’s *post hoc* test was performed to determine pairwise differences between band colors. Pearson’s correlation coefficients were calculated to evaluate the strength of the relationship between band elongation and generated force for each ERB color. Statistical analyses were performed using XLSTAT 2023.4 (Addinsoft, Paris, France).

## 3 Results

**Figure 5:**
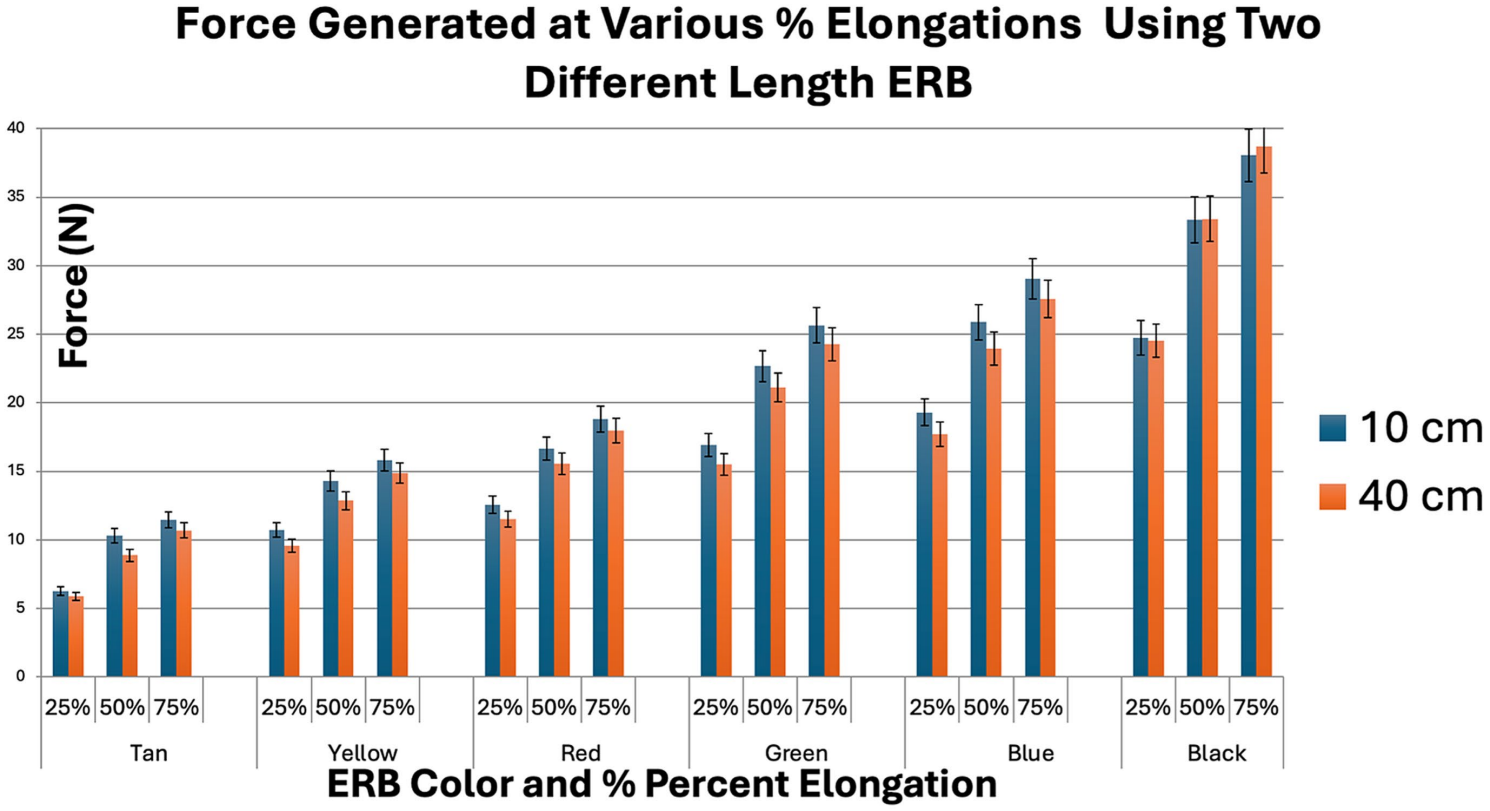
Force generated at various ERB elongation using 10 cm and 40 cm length ERB.

**Figure 6:**
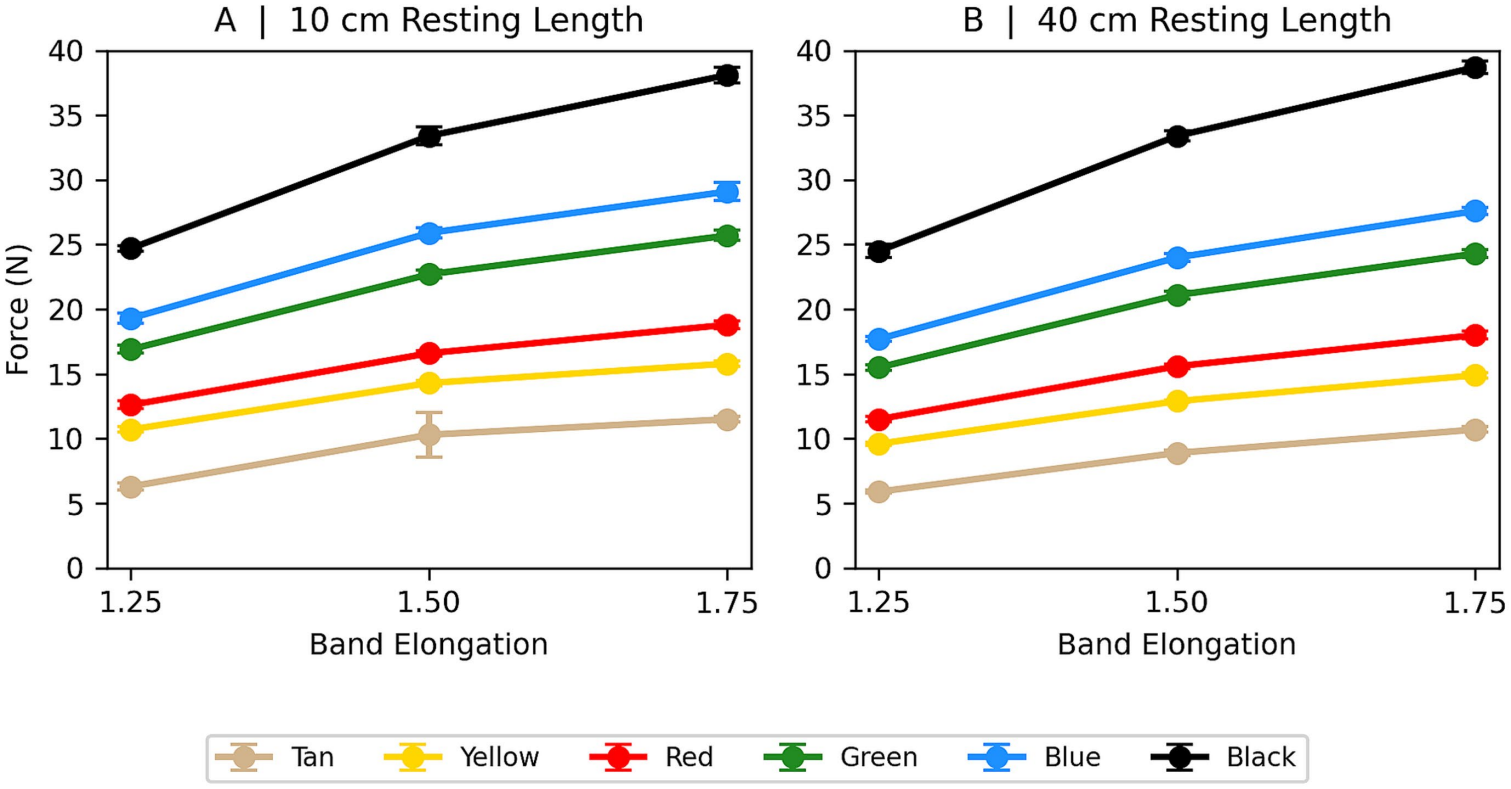
Mean tensile forces (± SD) produced by ERBs at elongation ratios of 1.25, 1.50, and 1.75 relative to resting length. **(A)** Bands tested at a resting length of 10 cm. **(B)** Bands tested at a resting length of 40 cm. Forces are reported in Newtons (N). Band colors correspond to commercially available TheraBand^®^ resistance levels.

The mean forces generated at each elongation level, expressed in newtons, are presented in Figure 5. The same data are additionally presented as kilograms-force (kg) to facilitate clinical interpretation and shown in Figure 6. A strong linear relationship between band elongation and generated force was observed for all ERB colors. Regression analysis yielded R^2^ values between 0.95 to 0.97(*p* < 0.05 for all bands; Table 1), confirming a highly consistent relationship between elongation and tensile force across bands. Following the preconditioning phase, peak force values remained stable across the 30 loading cycles for all band colors and lengths, with no observable drift in force generation over successive cycles.

**Table 1:** Calculated linear equations of elongation for each ERB color and resulting Pearson’s correlation coefficient.

TheraBand^®^ color	Linear equation	*R*^2^ value
Tan	*y* = 10.0× + 3.9	0.95
Yellow	*y* = 10.7× + 7.8	0.96
Red	*y* = 12.7× + 9.1	0.97
Green	*y* = 17.5× + 12.3	0.97
Blue	*y* = 19.6× + 14.1	0.97
Black	*y* = 27.4× + 18.4	0.97

All colors resulted in strong correlation to a best fit line.

In addition to the linear force generation characteristics of each ERB color, darker (stiffer) bands produced progressively greater resistance. At the maximum elongation tested (1.75× resting length), black bands generated approximately four times greater force than tan bands, illustrating the direct relationship between material stiffness and force output.

When comparing band lengths, the 40 cm bands consistently produced significantly lower mean forces than the corresponding 10 cm bands across all elongation levels (Table 2). This finding supports an inverse relationship between initial resting length and tensile force output: shorter bands yielded greater resistance than longer bands of the same color at a given elongation. This effect was evident across all color categories, reinforcing the combined influence of material stiffness and initial length on resistance magnitude.

**Table 2:** Mean forces (*N* ± standard deviation) produced using different ERB lengths, various elongation values, and different ERBs.

ERB color	Resting length (cm)	Elongation value compared to resting band length (force values are *N* ± SD with equivalent kg values shown for clinical reference)					
		1.25× (*N*)	1.25× (kg)	1.5× (N)	1.5× (kg)	1.75× (N)	1.75× (kg)
Tan	10	6.3 (± 0.3^a,*^)	0.642 (± 0.031^a,*^)	10.3 (± 1.7^g,†^)	1.05 (± 0.173^g,†^)	11.5 (± 0.2^m,#^)	1.173 (± 0.02^m,#^)
	40	5.9 (± 0.1^A,*^)	0.602 (± 0.01^A,*^)	8.9 (± 0.02^G,†^)	0.908 (± 0.02^G,†^)	10.7 (± 0.2^M,#^)	1.091 (± 0.02^M,#^)
Yellow	10	10.7 (± 0.2^b,*^)	1.091 (± 0.02^b,*^)	14.3 (± 0.2^h,†^)	1.458 (± 0.02^h,†^)	15.8 (± 0.2^n,#^)	1.611 (±0.02^n,#^)
	40	9.6 (± 0.1^B,*^)	0.979 (± 0.01^B,*^)	12.9 (±0.1^H,†^)	1.315 (±0.01^H,†^)	14.9 (± 0.2^N,#^)	1.519 (± 0.02^N,#^)
Red	10	12.6 (± 0.3^c,*^)	1.285 (± 0.031^c,*^)	16.6 (± 0.2^i,†^)	1.693 (± 0.02^i,†^)	18.8 (± 0.3^o,#^)	1.917 (±0.031^o,#^)
	40	11.5 (± 0.2^C,*^)	1.173 (± 0.02^C,*^)	15.6 (± 0.2^I,†^)	1.591 (± 0.02^I,†^)	18 (± 0.3^O,#^)	1.835 (±0.031^O,#^)
Green	10	16.9 (± 0.3^d,*^)	1.723 (± 0.031^d,*^)	22.7 (± 0.3^j,†^)	2.315 (± 0.031^j,†^)	25.7 (± 0.4^p,#^)	2.621 (± 0.041^p,#^)
	40	15.5 (± 0.2^D,*^)	1.581 (± 0.02^D,*^)	21.1 (± 0.3^j,†^)	2.152 (± 0.031^j,†^)	24.3 (± 0.3^P,#^)	2.478 (± 0.031^P,#^)
Blue	10	19.3 (± 0.4^e,*^)	1.968 (± 0.041^e,*^)	25.9 (± 0.4^k,†^)	2.641 (± 0.041^k,†^)	29.1 (± 0.7^q,#^)	2.967 (± 0.07^q,#^)
	40	17.7 (± 0.2^E,*^)	1.805 (± 0.02^E,*^)	24.0 (± 0.3^K,†^)	2.447 (± 0.031^K,†^)	27.6 (± 0.3^Q,#^)	2.814 (± 0.031^Q,#^)
Black	10	24.7 (± 0.2^f,*^)	2.519 (± 0.02^f,*^)	33.4 (± 0.7^l,†^)	3.406 (± 0.071^l,†^)	38.1 (± 0.6^r,#^)	3.885 (± 0.061^r,#^)
	40	24.5 (± 0.5^F,*^)	2.498 (± 0.051^F,*^)	33.4 (± 0.4^L,†^)	3.406 (± 0.041^L,†^)	38.7 (±0.5^R,#^)	3.946 (± 0.051^R,#^)

In addition, equivalent kg values are shown for clinical reference. Superscript lowercase letters indicate significant differences among colors of 10 cm bands within columns. Superscript uppercase letters indicate significant differences among colors with 40 cm bands within columns. Superscript symbols indicate significant differences among colors and elongation lengths within rows.

Clinical context of the generated data is important and one of the goals of the study reported here. Based on the data generated in our study, clinicians can select various ERBs, ERB lengths, and elongation amounts to apply specific forces for rehabilitation (Table 3).

**Table 3:** Chart for veterinary ERB dosing.

Elastic resistance band condition						
Force	Tan	Yellow	Red	Green	Blue	Black
1 kg force	10 cm, 1.5× stretch	10 cm, 1.25× stretch	40 cm, 1.25× stretch			
	40 cm, 1.75× stretch	40 cm, 1.25× stretch				
1.5 kg force		10 cm, 1.5× stretch 40 cm, 1.75× stretch	40 cm, 1.5× stretch	40 cm, 1.25× stretch		
2 kg force			10 cm, 1.75× stretch	40 cm, 1.5× stretch	10 cm, 1.25× stretch	
2.5 kg force				10 cm, 1.75× stretch	10 cm, 1.5× stretch	10 cm, 1.25× stretch
				40 cm, 1.75× stretch	40 cm, 1.5× stretch	40 cm, 1.25× stretch
3 kg force				10 cm, 1.75× stretch	10 cm, 1.75× stretch	
3.5 kg force						10 cm, 1.5× stretch
						40 cm, 1.5× stretch
4 kg force						40 cm, 1.75× stretch

## 4 Discussion and conclusions

The observed linear elastic behavior of all tested ERBs is consistent with Hooke’s Law and aligns with previously published data from biomechanical studies of ERBs primarily involving human applications (10, 15). Our results showed a proportional increase in force with increasing elongation for each band, similar to prior reports of ERB materials exhibiting linear force-elongation relationships after initial slack is overcome (15). However, the absolute force values recorded in this veterinary-oriented study were lower than those typically reported in human applications. For instance, one Thera-Band^®^ study indicated that force at 175% elongation (30 cm resting length) was approximately 46 N for the black THERABAND^®^ (10), whereas our highest measured force at 175% elongation (10 and 40 cm resting lengths) was approximately 38 N. That same study revealed that almost all eight bands measured much less than those predicted by the manufacturer (THERABAND^®^), which could lead to overestimation of the prescribed resistance (10).

As hypothesized, the stiffer (darker) bands and increased elongation produced increased tensile forces, although shorter band segments exhibited more resistance compared to longer segments of identical material. This affirms that band stiffness, elongation, and beginning length are critical factors influencing applied resistance. Clinically, these relationships are crucial. Understanding how changes in band selection and stretch length affect resistance forces enables therapists to design more precise, reproducible, and individualized exercise prescriptions. Our results emphasize the need for standardized ERB selection and stretching parameters to ensure consistent mechanical loading across patients and sessions. If not controlled, dosing may result in inconsistencies, leading to either inadequate muscle activation or excessive tensile forces that may result in harm to vulnerable patients such as in the early postoperative period or with neurological compromise (16).

Species-specific factors may also result in differences in use of ERBs for strengthening, such as differences in joint excursion, altered stride lengths of dogs, and placement of the ERBs on various portions of the limbs, which may result in different limb moment arms in comparison to humans (17). Such biomechanical differences emphasize the need for species-specific calibration when applying elastic resistance-based exercises. Force profiles established in human contexts may not apply to veterinary patients without considerations of these factors and adjustments.

In one recent cross-over study, 29 young healthy human adults were used to test the effectiveness of ERBs compared to conventional resistance equipment (barbells or cable machines). They found that ERBs underperform when the bands are slack (beginning of the motion) but are comparable when stretched (end range) (18).

Subsequent studies should seek to corroborate these *in vitro* results in live canines, linking mechanical force output to physiological responses including electromyographic muscle activation, joint kinematics, and functional rehabilitation outcomes. Predictive models, exemplified in Figure 6, can be utilized to estimate the forces exerted during different ERB exercises. These findings provide quantitative data on ERB force generation under conditions commonly used in canine rehabilitation and offers a practical guideline for precise exercise dosing and enhance personalized rehabilitation regimens in veterinary practice.

### 4.1 Clinical implications

The findings of this study provide clinicians with a practical framework for prescribing resistance-based therapeutic exercises using ERBs. By understanding the predictable relationship between band color (stiffness), elongation, and length, veterinarians and physical therapists can accurately titrate band stretching to achieve desired resistance levels. For instance, if approximately 2 kg of resistance is the target for a canine stifle extension exercise, one could select a 10 cm blue band and stretch it to 1.25 × its resting length (based on our force-elongation data), a 40 cm green band stretched to 1.5× its resting length, or a 10 cm red band stretched to 1.75× its resting length (Table 3). When higher resistance is required, such as during advanced strengthening or late-stage rehabilitation, practitioners can adjust the parameters by using a shorter band segment or a stiffer (darker-colored) band to increase the force output.

We have provided an accompanying force–elongation graph (Figure 6) and a reference chart of force (in kg) for each band color at various elongation lengths (Table 3). These tools allow for quick selection of a band–elongation combination to match a specific target force. Implementing such data promotes individualized rehabilitation dosing, enhances reproducibility between sessions, and helps ensure consistent loading across different patients. With appropriate calibration, the principles demonstrated here for canine patients can be extended to other veterinary species and even adapted to human rehabilitation exercises that use elastic resistance training.

Although tensile force produced by the band represents the primary mechanical parameter measured in this study, the effective load applied to a joint during rehabilitation exercises will depend on additional biomechanical factors including band attachment location, limb position, and resulting moment arm about the joint (13). Consequently, the force values reported here should be interpreted as the resistance generated by the band itself rather than the exact joint torque experienced by the patient.

### 4.2 Limitations and future work

This study has several limitations. First, only a single commercial brand of ERB (THERABAND^®^) was evaluated. Although five bands of each color were tested, all bands were obtained from the same manufacturer and production batch. Variability in manufacturing between bands or production batches may affect the mechanical characteristics and resistance profiles of elastic materials (10, 19); however, assessing this is outside the purview of this study. Subsequent research should assess several bands across distinct production lots to delineate potential variability more accurately. Future studies should evaluate multiple bands across different production lots to better characterize potential variability.

Second, mechanical testing was performed *in vitro* under controlled laboratory conditions using a biomechanical testing system. In a clinical rehabilitation setting, factors such as limb position, anchor point height, joint angles, movement velocity, and attachment method may influence the actual resistance experienced by the patient and affect results. For example, in our clinical experience, dogs often will not advance their limb if the resistance is too great, especially at higher speeds, such as a trot compared to a walk (3).

Third, although bands were pre-conditioned prior to testing to reduce viscoelastic effects, elastic resistance materials can be subject to material factors such as temperature, band age, cumulative creep and fatigue (19, 20) from repeated loading cycles. While peak forces remained stable across testing cycles in this study, prolonged clinical use of ERBs may alter their mechanical properties over time and should be evaluated in future studies.

Finally, greater variability was observed in some conditions, particularly with the 40 cm red bands. This may reflect minor differences in material uniformity or slight alignment variability during testing, which may become more apparent when longer segments of elastic material are evaluated. However, this increased variability did not affect the statistical significance and consistency of results found with other bands. Additional studies evaluating larger sample sizes of bands may help further characterize these sources of variability. We reported only mechanical force outputs and did not evaluate patient outcomes, so the translation of these force levels to functional improvement remains to be validated *in vivo*.

Future work should (1) test additional band brands and lengths, (2) model viscoelastic effects of repeated use over time, (3) validate the predictive force equations against in-clinic measurements, and (4) link prescribed forces to patient-centered outcomes to refine evidence-based rehabilitation dosing guidelines.

## Data Availability

The raw data supporting the conclusions of this article will be made available by the authors, without undue reservation.
